# Recurrent retroperitoneal Schwannomas displaying different differentiation from primary tumor: Case report and literature review

**DOI:** 10.1186/1477-7819-8-66

**Published:** 2010-08-09

**Authors:** Zhong-qi Li, Hai-yong Wang, Jun Li, Lisong Teng

**Affiliations:** 1 Cancer Center, The First Affiliated Hospital, Zhejiang University School of Medicine, Hangzhou, China; 2 Department of Pathology, The First Affiliated Hospital, Zhejiang University School of Medicine, Hangzhou, China

## Abstract

**Background:**

Retroperitoneal Schwannomas are uncommonly found in the retroperitoneum and few of them show malignant transformation and invasion. Local recurrence are common in malignant Schwannomas with very few reports of tumor distinct differentiation at recurrences.

**Case presentation:**

We report here a rare case of retroperitoneal schwannoma with multiple origins from retroperitoneum and pelvic wall. Pathological examination confirmed the case as a schwannoma with malignant transformation. Radical dissection of the tumors along with the sacrifice of adjacent sigmoid colon and left kidney failed to provide a cure for this patient. Due to tumor recurrence, a second and a third surgery of radical excision were performed 6 months and 17 months later after the first surgery, respectively. Histopathologic analysis identified that the recurrent tumors were different from the original schwannoma because of their smooth muscle-like differentiation.

**Conclusion:**

Malignant schwannomas are uncommon sarcomas with a high incidence of local recurrence. Distinct immunohistochemical staining results of the tumors at recurrence indicate their potential of smooth-muscle like differentiation. Radical excision of the tumors may provide benefit for their local recurrences.

## Background

Schwannomas are a rare variant of peripheral nerve sheath tumors that seldomly develop to malignancy. In the absence of Recklinghausen's disease, these masses rarely occur in the retroperitoneum, which has a reported incidence of only 0.5-5% of all schwannomas [1,2]. Schwannomas usually occur as solitary encapsulated tumors with demarcated margins with neighbour organs. We report our experience with a rare case of malignant transformed schwannoma invading kidney and sigmoid colon at its first occurrence. Tumors recurred after complete resection along with adjacent tissue and viscera. Interestingly, tumors showed smooth-muscle like differentiation at recurrences.

## Case Presentation

**Figure 1:**
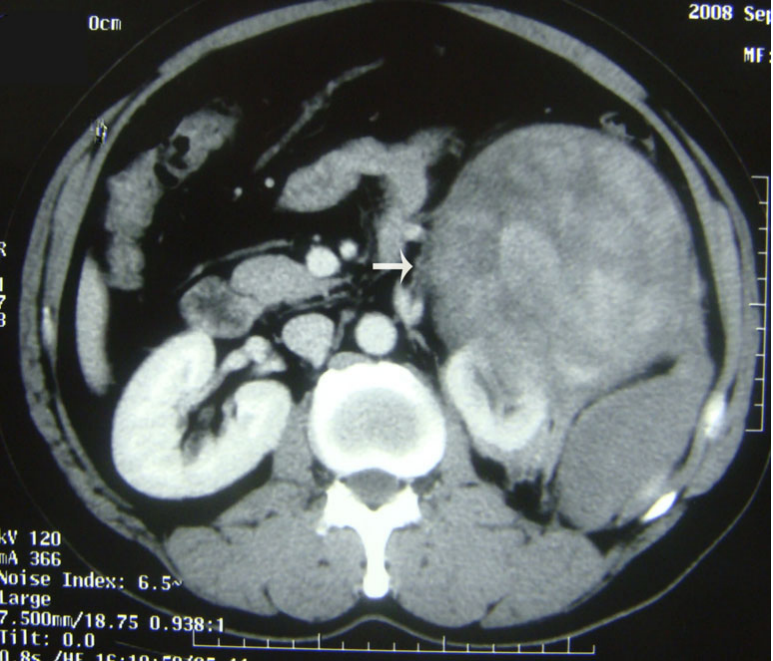
**Computed tomography (CT) showing a giant mass located in the retroperitoneal space with invasion to the left kidney**.

**Figure 2:**
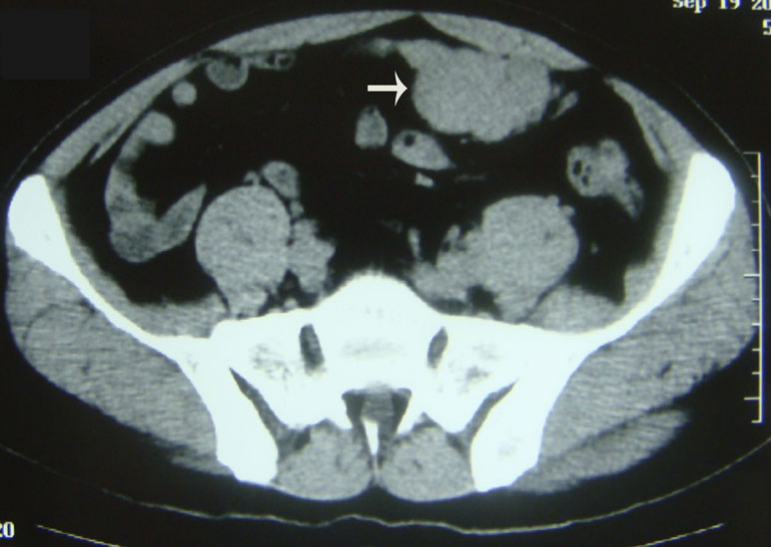
**CT scan showing tumors involved the sigmoid flexure**.

**Figure 3:**
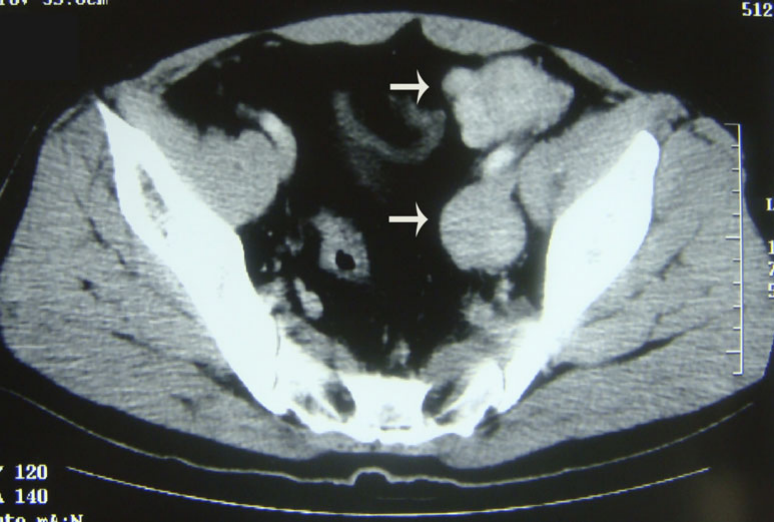
**CT scan showing smaller masses located in the pelvis cavity**.

A large mass in the left lower quadrant of the abdomen was found in a 51 year old male patient during physical examination and confirmed by computer tomography. The patient has no history of any cancer and no family history of neurofibromatosis. Lab tests results including blood routine, urine routine, serum chemistry, as well as examinations of several serum tumor markers such as Carcinoembryonic antigen (CEA), α-fetoprotin (AFP) and Carbohydrate antigen 19.9 (CA19.9) were all within normal ranges. Computed tomography scan confirmed a 15.0 × 12.3 × 10.0-cm enhancing heterogeneous mass with left kidney invasion arising from retroperitoneal space (Fig. 1). As shown in the CT scan, we could identify several smaller nodular lesions around the main tumor, locating in the soft tissues of the pelvic wall and sigmoid colon (Fig. 2 and Fig. 3). A presumptive diagnosis of retroperitoneal sarcoma or possible pancreatic neoplasm was suspected. The patient underwent surgical excision of the masses. The largest retroperitoneal mass was speculated to be the original site upon examinations during the operation. The nodules found on the sigmoid colon were most likely "satellite" lesions, for it was confined in the colon wall but did not protrude into the colon cavity, which may due to direct spread of tumor cells from the primary tumor. However, the pelvic masses rooted from the pelvic wall but not from pelvic peritoneum indicated they may have independent origins. The left kidney and sigmoid flexure were extirpated together with the tumors for the negative soft tissue margins. No further anti-tumor therapy was administrated after the surgery, and CT scan of the abdomen and pelvic cavity was performed every three months as follow-up. In the follow-up examination 6 months post surgery, a mass 7-cm in diameter was discovered at the retroperitoneal site (Additional file 1: Fig. S1). The mass was revealed to be well-circumscribed and complete excision was performed. The patient was doing well after the second surgery till 11 months after, the second surgery, when a recurrent mass involving the splenic hilum and cauda pancreatic was revealed by CT. A third surgery was performed to resect the tumor together with the spleen and distal pancreas. All of the three surgeries were performed to obtain macroscopic clearance at resection.

### Pathological findings

**Figure 4:**
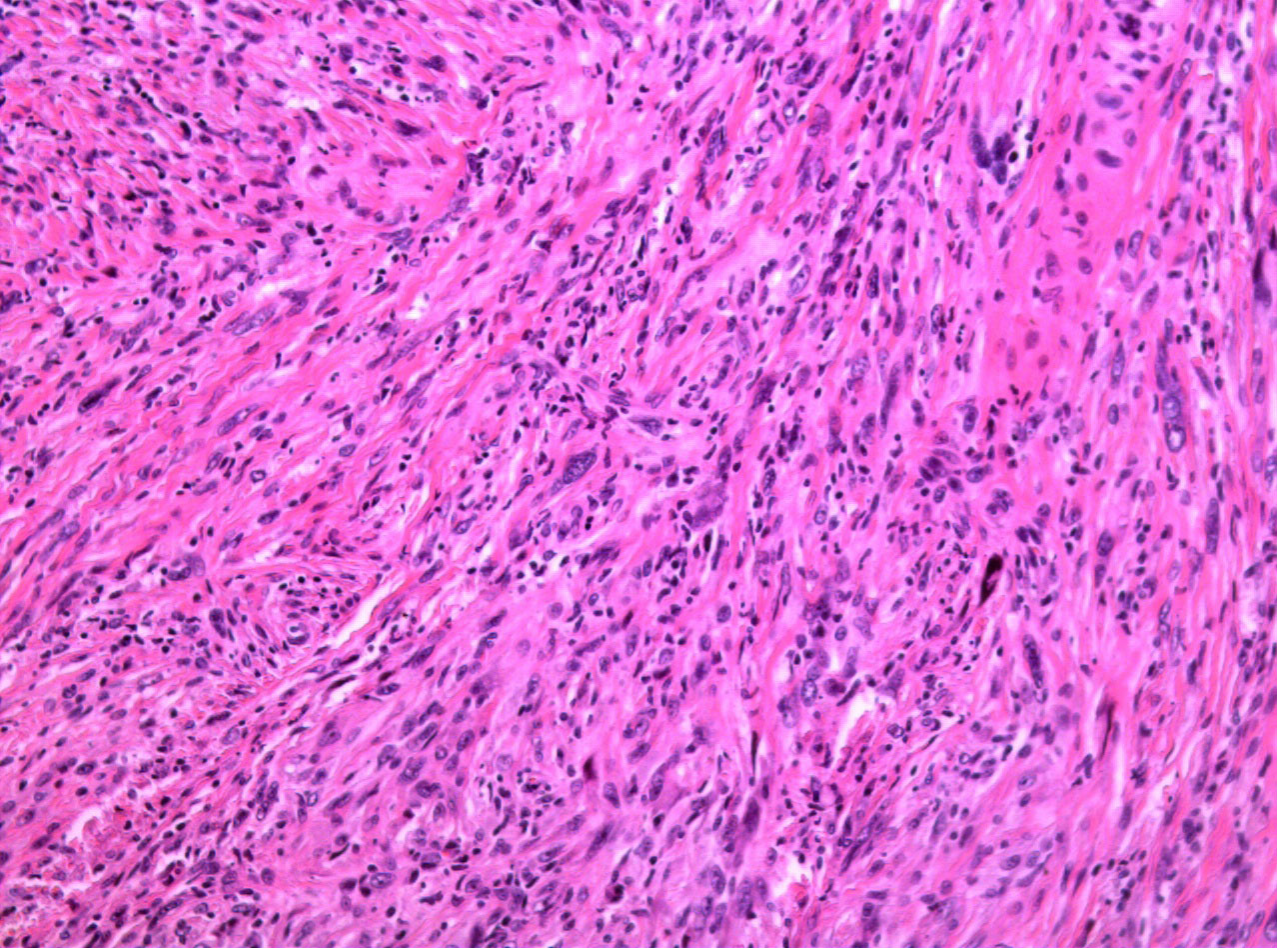
**Images of HE staining showing the schwannoma undergoing malignant transformation**. High-power view of the spindle cell component showing bland cytologic features with a suggestion of palisading, mitotic figures and tumor giant cells. (Original magnification 200×).

**Figure 5:**
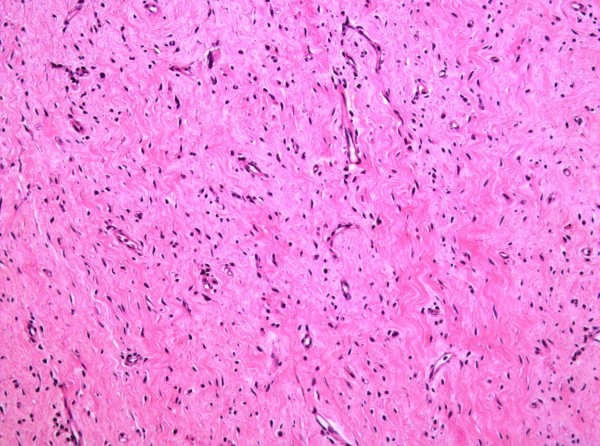
**Benign part of the tumor showing hypocellular area without features of cytologic atypia**. (Original magnification 200×).

At first occurrence, the biggest primary mass had a grey fish-meat like appearance on section with major solid features. Histopathological analysis identified the tumor as a schwannoma with malignant transformation. It was not grossly clear whether the lesion was associated with a nerve trunk. Histologically, the lesion consisted of spindle cells arranged in short bundles with occasional palisading nuclei. Cytologic atypia is evident as characterized by nuclear hyperchromasia, atypical mitotic figures and tumor giant cells (Fig. 4 and Fig. 5). Seromuscular layer of sigmoid colon and renal capsule were invaded by tumor cells as shown in HE staining section (Additional file 2: Fig. S2 and Additional file 3: Fig. S3). All the smaller nodular lesions on the colon and pelvic cavity had the same histopathological characters with the largest one and the immunohistochemical profile were very similar.

**Figure 6:**
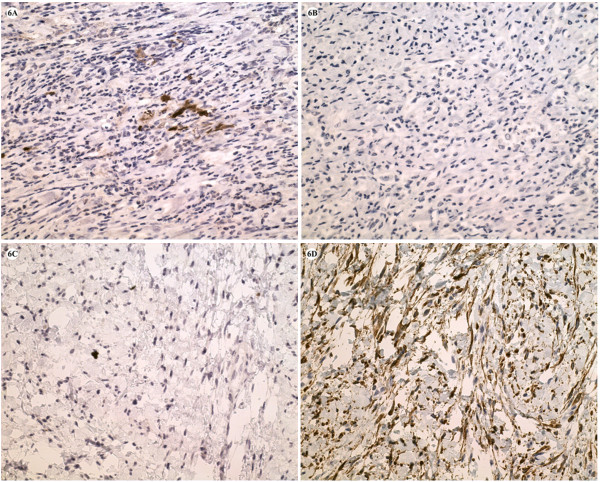
**Immunohistochemical features of S-100 and desmin staining in the primary retroperitoneal mass (A and B) and recurrent masses (C and D)**. The spindle cells of the largest primary mass were positive for S-100 protein (1+) (A) and negative for desmin (B). The spindle cell component of the recurrent mass was negative for S-100 staining (C), while showed strong immunoreactivity to desmin (D). (Original magnification200×).

Immunohistochemical studies were performed using a panel of antibodies, including S-100, Desmin, CK, EMA, CD117 and SMA (Dako, CA, USA). All specimens from the three surgical resection were negative for CK, EMA, CD117 and SMA staining. Interestingly, the spindle cells in the first resected specimen were positive for S-100 (Fig. 6A) but negative for Desmin (Fig. 6B). In contrast, specimens from the later two operation were negative for S-100 (Fig. 6C) but strongly positive for desmin (Fig. 6D). These findings suggested diverse differentiation of the schwannoma at recurrences.

## Discussion

Most previously reported cases of retroperitoneal schwannomas were solitary tumors except for a few reports of multiple tumors cases [3]. Our case showing multiple tumors at different location simultaneously in the absence of neuromatosis background was quite rare. In Voros's study, 7/29 cases presented with small nodular lesions around the main retroperitoneal tumor, which turn out to be satellite tumors, having an adverse effect on recurrence and survival rates [4]. The nodules found on the sigmoid colon in our case most likely represent "satellite" lesion, which may due to direct spread of tumor cells from the primary tumor. However, the pelvic ones were shown to be originated from tissues below the pelvic peritoneum, which indicated these tumors are of independent origins rather than metastasis of the retroperitoneal tumor.

Very few Schwannomas were reported to undergo malignant transformation and invade adjacent viscera, including colon and kidney [5]. Although diffuse positivity for S100 protein in the cytoplasm of tumor cells is the classical immunohistochemical staining feature of benign schwannomas, the variants with malignant degeneration lesion may vary. The diagnosis of malignant peripheral nerve sheath tumor lacks standardized diagnostic criteria except that features such as dense fascicules in a "marble-like" pattern consisting of asymmetrically tapered spindle cells [1]. Malignancy is usually characterized histologically by mitosis figures, pleomorphism, and blood vessel infiltration [6].

Schwannomas have been reported to be coexisted with focal smooth muscle differentiation [7]. Controversy still exists in the pathological diagnosis of the recurrent retroperitoneal tumors. The cellular areas in the second surgical specimen showed strong desmin expression but not S-100. In view of this expression pattern, a diagnosis of schwannoma was felt to be less likely and a report of low-grade sarcoma, possibly showing smooth muscle differentiation was issued. However, distinct immunohistochemical staining results in our case might also indicate different directions of differentiation of the malignant schwannoma. One possible explanation for this observation may be the schwannoma was derived from multipotential neural cells that could have developed into various phenotypes. This theory can also explain the different types of elements found in the schwannoma [8,9].

Malignant schwannomas are aggressive tumors that act as high-grade sarcomas with a high incidence of local recurrence or distant metastasis [10]. Recurrence, however, has been reported to occur usually within 6 months of the initial surgical treatment [11]. The rates of local recurrence range from 16% to 54% after conservative intralesional enucleation [12]. According to a recent view of the surgical management of primary retroperitoneal sarcomas, the completeness of resection and tumor grade are the most important predictors of local recurrence and overall survival [13]. The prognosis of this schwannomas with malignant transformation is correlated with tumor location, degree of differentiation, adjacent invasion and thoroughness of surgical excision [1,14], however, to the best of our knowledge, the risk of recurrence for this type of rare tumors has not yet been systematically studied. In our case, the reason of repeated recurrences within a relatively short period is unknown, we wonder whether multiple origins and evident malignancy features such as atypical mitosis, giant tumor cell, local organ invasion may be two of the risk factors.

Radical excision is considered to be the best way to treat retroperitoneal neural sheath tumors, however, considerable controversy exists concerning the soft tissue margins that are negative of tumor invasion [1]. Some authors argued for complete surgical excision that may include, if necessary, the sacrifice of adjacent tissue and viscera [15,16]. Others believe that since schwannoma is usually a benign mass, a simple enucleation or partial excision of the tumor is sufficient [17,18]. Re-operation may provide a cure for the locally recurred schwannoma [19]. Nevertheless, in our case, although a complete excision of the tumor with the clear adjacent tissue margins was executed in the first operation, recurrence occurred within 6 months and a second radical excision operation could not prevent its recurrence at the same location for a third time. Since adjuvant radiotherapy and chemotherapy did not appear to provide any proven benefit and the single most important prognosis factor is aggressively successful en bloc resection of the primary tumor, no adjuvant therapy after surgery was given in our case [20]. The patient is currently doing well 6 months after the third surgery, however, long-term follow-ups are warranted for this rare case of multiple and recurrent schwannoma.

## Conclusion

Malignant schwannomas are uncommon sarcomas with a high incidence of local recurrence. Distinct immunohistochemical staining results of the tumors at recurrence indicate their potential of smooth-muscle like differentiation. Radical excision of the tumors may provide benefit for their local recurrences.

## Consent

Written informed consents were obtained from the patient for publication of this case report and accompanying images. Copies of the written consent are available for review upon request.

## Competing interests

The authors declare that they have no competing interests.

## Authors' contributions

WHY wrote the initial draft. LZQ and WHY contributed equally to this work. TLS is the guarantor. All authors read and approved the final manuscript.

## Funding support

This study was partly supported by National Basic Research Program of China (973 Program, No.2009CB521704).

## Supplementary Material

Additional file 1 **Fig S1. Recurrent schwannoma**. Schwannoma recurred at the retroperitoneal space 6 months later after the first surgery as indicated by arrow. Click here for file

Additional file 2 **Fig S2. Tumor invading colon**. Pathological findings showed the tumor invasion to the colon seromuscular layer. (Original magnification 200×). Click here for file

Additional file 3 **Fig S3. Tumor invading kidney**. Pathological findings showed the tumor invasion to renal capsule. (Original magnification 200×). Click here for file

## References

[B1] Cury J Coelho RF Srougi M Retroperitoneal schwannoma: case series and literature review Clinics 2007 62 359 62 17589680 10.1590/s1807-59322007000300024

[B2] Li Q Gao C Juzi JT Hao X Analysis of 82 cases of retroperitoneal schwannoma ANZ J Surg 2007 77 237 40 10.1111/j.1445-2197.2007.04025.x 17388825

[B3] Hurley S Smith JJ Larsen CR Silverman ML Multiple retroperitoneal schwannomas: case report and review of the literature J Urol 1994 151 413 6 8283540 10.1016/s0022-5347(17)34966-2

[B4] Voros D Theodorou D Ventouri K Prachalias A Danias N Gouliamos A Retroperitoneal tumors: do the satellite tumors mean something? J Surg Oncol 1998 68 30 3 10.1002/(SICI)1096-9098(199805)68:1<30::AID-JSO7>3.0.CO;2-M 9610660

[B5] Enzinger FM Weiss SW Enzinger FM, Weiss SW Benign tumors of peripheral nerves Soft Tissue Tumours 1995 3 St Louis: Mosby 821 888

[B6] Chen KT Latorrace R Fubich D Padgug A Hafez G Gilbert EF Malignant schwannoma: A light microscopy and ultrastructural study Cancer 1980 45 1583 1593 10.1002/1097-0142(19800401)45:7<1585::AID-CNCR2820450712>3.0.CO;2-P 7370917

[B7] Anderson CE Salter DM Schwannoma with focal smooth muscle differentiation: a potential pitfall in the interpretation of core biopsies Histopathology 2005 46 592 594 10.1111/j.1365-2559.2005.02020.x 15842646

[B8] Ducatman BS Scheithauer BW Malignat peripheral nerve sheath tumors with divergent differentiation Cancer 1984 54 1049 1057 10.1002/1097-0142(19840915)54:6<1049::AID-CNCR2820540620>3.0.CO;2-1 6432304

[B9] Chuang ST Wang HL An unusual case of glandular schwannoma Hum Pathol 2007 38 673 677 10.1016/j.humpath.2006.10.016 17258283

[B10] Takatera H Takiuchi H Namiki M Takaha M Ohnishi S Sonoda T Retroperitoneal schwannoma Urology 1986 28 529 531 10.1016/0090-4295(86)90161-5 3787928

[B11] White HR Survival in malignant schwannoma Cancer 1971 27 720 729 10.1002/1097-0142(197103)27:3<720::AID-CNCR2820270331>3.0.CO;2-D 5549502

[B12] Andonian S Karakiewicz PI Herr HW Presacral cystic schwannoma in a man Urology 2003 62 8 10 10.1016/S0090-4295(03)00481-3 12946771

[B13] Strauss DC Hayes AJ Thway K Moskovic EC Fisher C Thomas JM Surgical management of primary retroperitoneal sarcoma Br J Surg 2010 97 698 706 20306527 10.1002/bjs.6994

[B14] Song JY Kim SY Park EG Kim CJ Kim DG Schwannoma in the retroperitoneum J Obstet Gynaecol Res 2007 33 371 375 10.1111/j.1447-0756.2007.00539.x 17578370

[B15] Daneshmand S Youssefzadeh D Chamie K Boswell W Wu N Stein JP Boyd S Skinner DG Benign retroperitoneal schwannoma: A case series and review of literature Urology 2003 62 993 997 10.1016/S0090-4295(03)00792-1 14665342

[B16] Girgin C Ozkan U Sezer A Tugyan N A large pelvic schwannoma causing bilateral hydronephrosis Int J Urol 2003 10 616 618 10.1046/j.1442-2042.2003.00702.x 14633089

[B17] Gubbay AD Moschilla G Gray BN Thompson I Retroperitoneal schwannoma: a case series and review Aust N Z J Surg 1995 65 197 200 10.1111/j.1445-2197.1995.tb00607.x 7887865

[B18] Regan JF Juler GL Schmutzer KJ Retroperitoneal neurilemoma Am J Surg 1977 134 140 145 10.1016/0002-9610(77)90297-5 879406

[B19] Abernathey CD Onoerio BM Scheithauer B Pairolero PC Shives TC Surgical management of giant sacral schwannomas J Neurosurg 1986 65 286 295 10.3171/jns.1986.65.3.0286 3734878

[B20] Pirayesh A Chee Y Helliwell TR Hershman MJ Leinster SJ Fordham MV Poston GJ The management of retroperitoneal soft tissue sarcoma: a single institution experience with a review of the literature Eur J Surg Oncol 2001 27 491 497 10.1053/ejso.2001.1146 11504522

