# Supplementary figures and images for: Recurrent retroperitoneal Schwannomas displaying different differentiation from primary tumor: Case report and literature review
Source: World J Surg Oncol. 2010 Aug 9;8:66. doi: 10.1186/1477-7819-8-66 (PMC2927596; doi:10.1186/1477-7819-8-66)

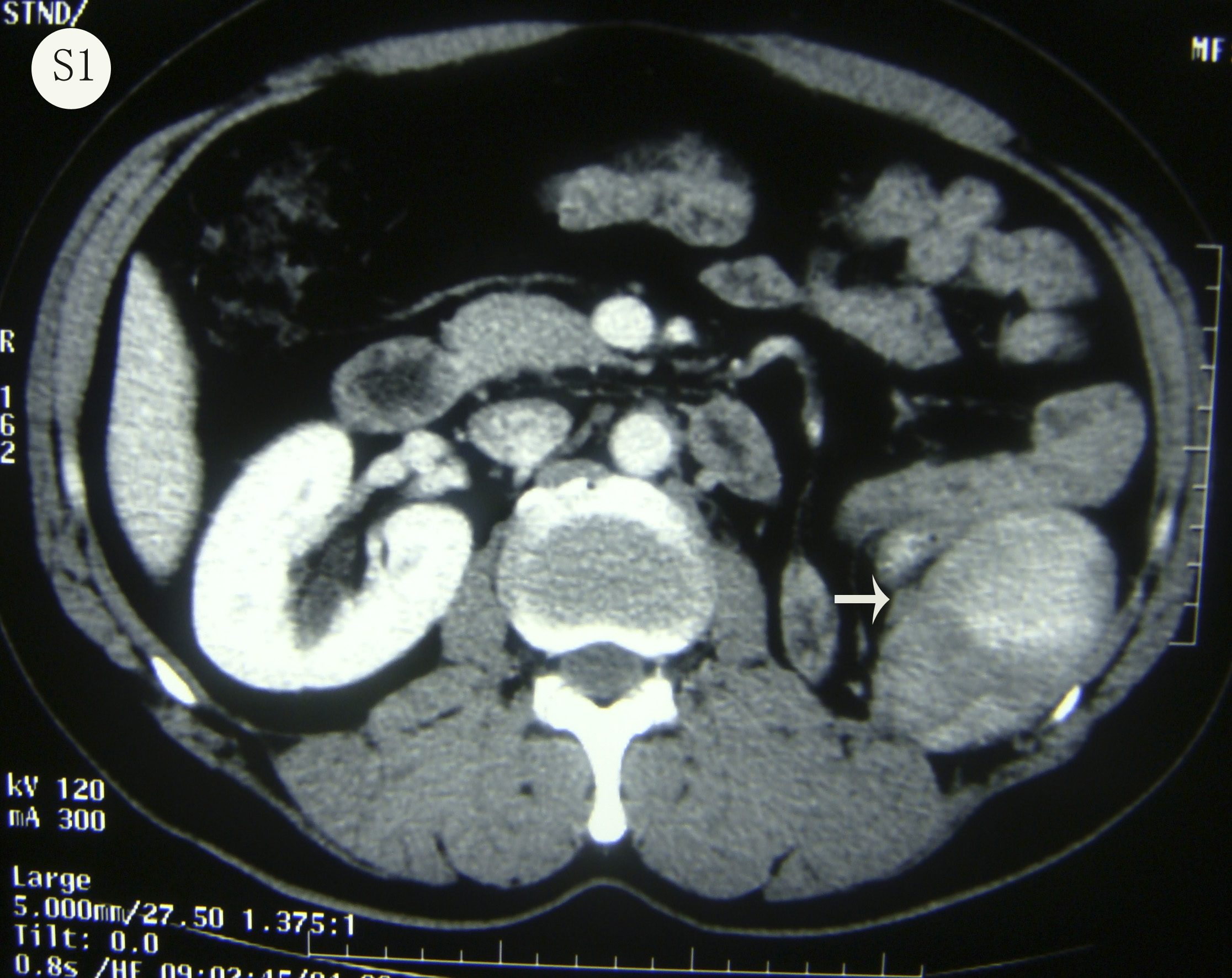

Supplement: Additional file 1 — Fig S1. Recurrent schwannoma. Schwannoma recurred at the retroperitoneal space 6 months later after the first surgery as indicated by arrow. [file 1477-7819-8-66-S1.JPEG]

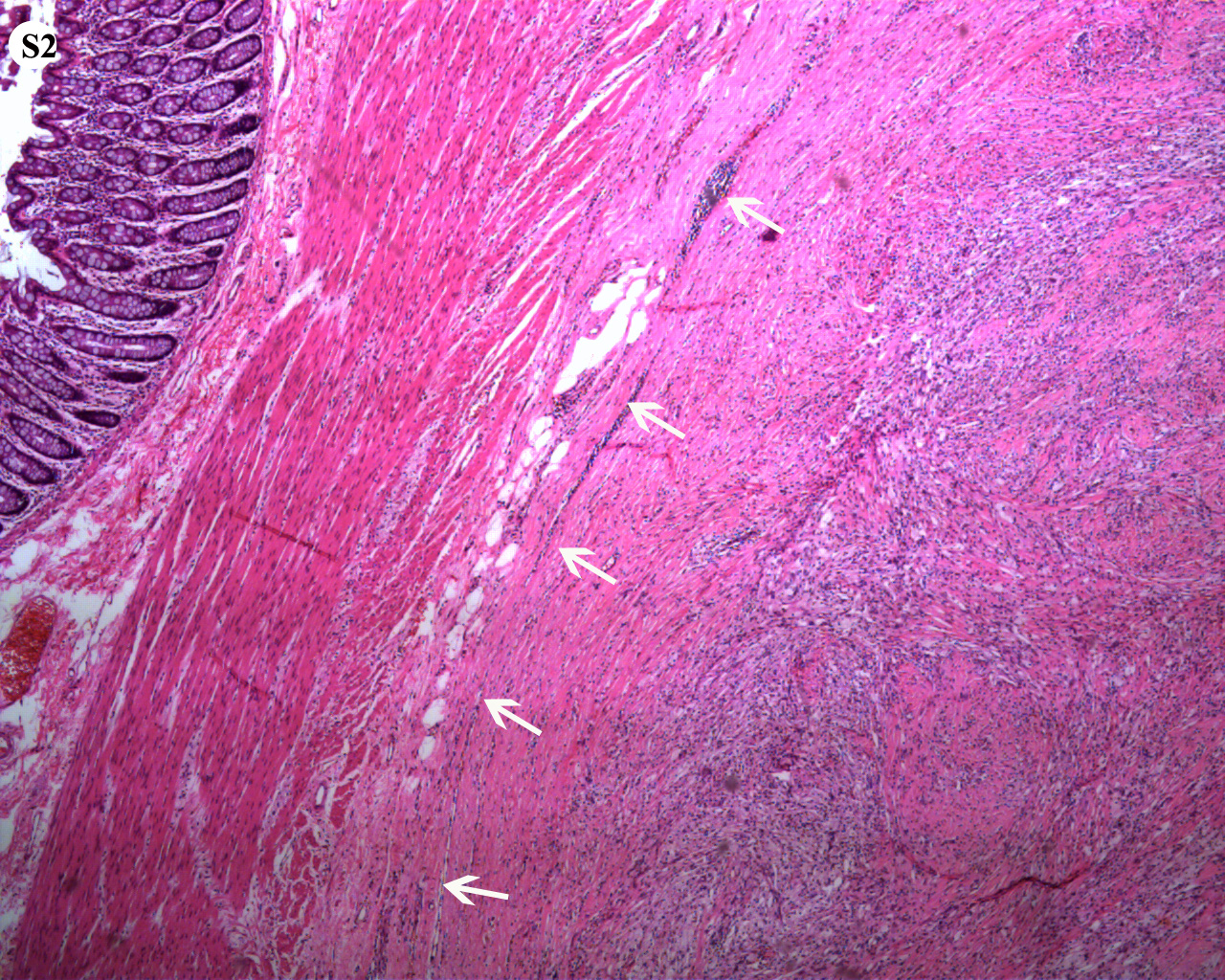

Supplement: Additional file 2 — Fig S2. Tumor invading colon. Pathological findings showed the tumor invasion to the colon seromuscular layer. (Original magnification 200×). [file 1477-7819-8-66-S2.JPEG]

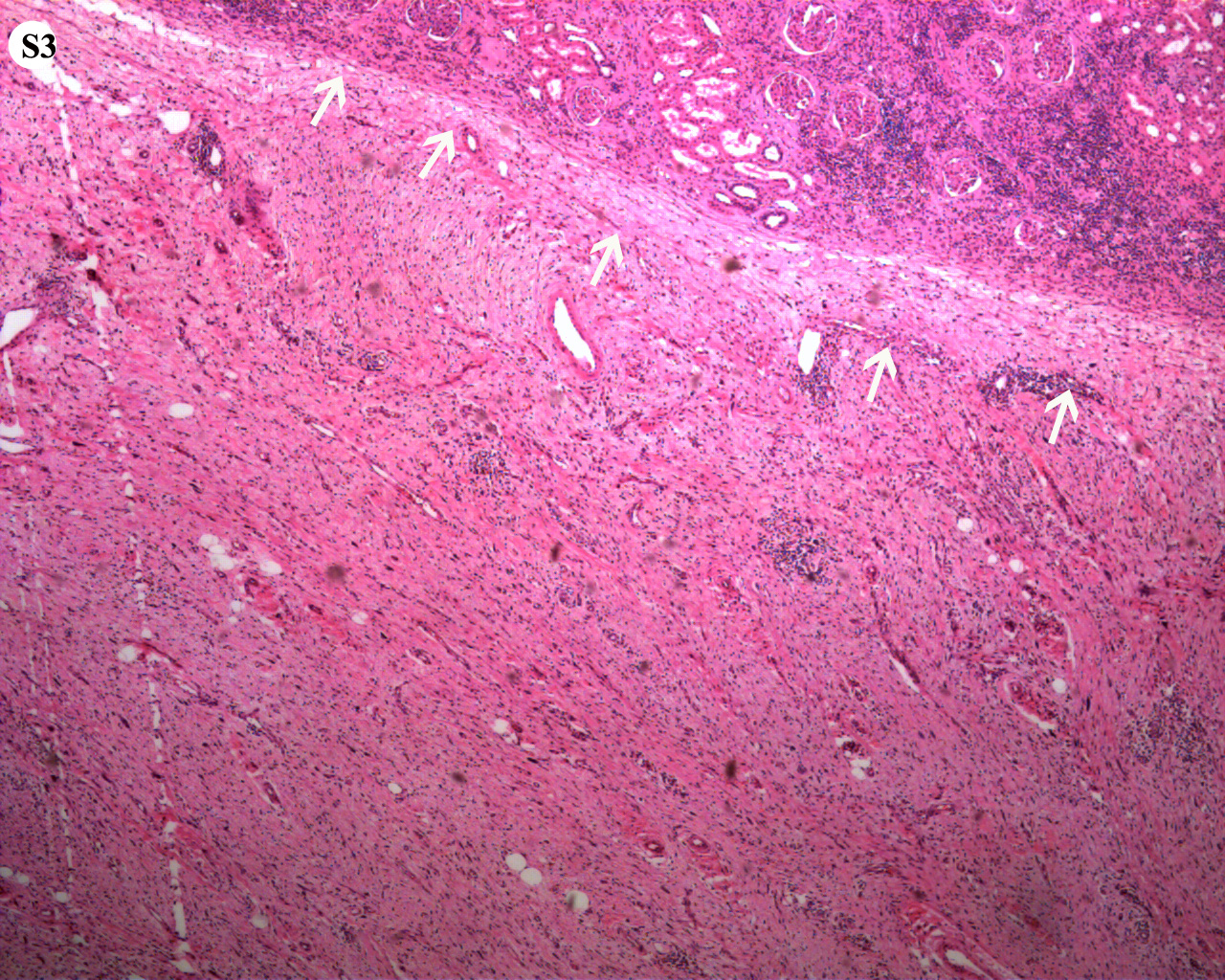

Supplement: Additional file 3 — Fig S3. Tumor invading kidney. Pathological findings showed the tumor invasion to renal capsule. (Original magnification 200×). [file 1477-7819-8-66-S3.JPEG]
